# Constitutional Delay Influences the Auxological Response to Growth Hormone Treatment in Children with Short Stature and Growth Hormone Sufficiency

**DOI:** 10.1038/srep06061

**Published:** 2014-08-14

**Authors:** Katherine C. Gunn, Wayne S. Cutfield, Paul L. Hofman, Craig A. Jefferies, Benjamin B. Albert, Alistair J. Gunn

**Affiliations:** 1 Department of Physiology, Faculty of Medical and Health Sciences, University of Auckland; 2 Clinical Endocrinology Group, Liggins Institute, University of Auckland; 3 Paediatric Endocrinology Service, Starship Children's Hospital, Auckland, New Zealand

## Abstract

In a retrospective, population based cohort study, we examined whether constitutional delay was associated with the growth response to growth hormone (GH) in children with short stature and normal GH responses. 70 patients were treated with 21 GH iu/m2/week from 1975 to 2013 throughout New Zealand. Demographic and auxological data were prospectively collected and standard deviation scores (SDS) were calculated for height (HtSDS), yearly growth velocity (GV-SDS), body mass index (BMI-SDS) and predicted adult height (PAH-SDS) at time of the last available bone age. In the first year, GH was associated with marked increase in HtSDS (+0.46 (0.19, 0.76), p < 0.001) and GV-SDS (from −1.9 (−3.6, −0.7) to +2.7 (0.45, 4.2), p < 0.001). The increase in HtSDS but not in GV-SDS was greatest with younger patients and greater bone age delay, with no effect of sex, BMI-SDS or baseline HtSDS. PAH-SDS increased with treatment (+0.94 (0.18, 1.5)); increased PAH-SDS was associated with less bone age delay and greater initial increase in HtSDS. This study shows that greater bone age delay was associated with greater initial improvement in height but less improvement in predicted adult heights, suggesting that children with very delayed bone ages may show accelerated maturation during GH treatment.

Idiopathic short stature (ISS) is a condition in which a child is short for unknown reasons and consistently grows at a below average rate for their demographic^1^,^2^. There is evidence from controlled trials that recombinant human growth hormone (GH) can increase short term growth rates and that this increases final height by a mean of approximately 5.5 cm^1^,^3^,^4^,^5^. Not surprisingly in this heterogeneous group, the response to GH is highly variable and some patients show little or no apparent gain in height. Previous studies suggest that better growth is associated with younger age at the start of GH treatment, taller height at start of GH therapy, taller parents and greater response to GH in the first year^1^,^4^,^6^. In Australia, a retrospective study suggested that girls had a reduced response compared to boys when GH was begun before 6 years of age, for unclear reasons^4^.

Constitutional delay, defined as a biological age a year or more behind the child's chronological age, is a measure of the potential future growth of the child^7^. Healthy children with ISS tend to have a delayed bone age. Perhaps surprisingly the difference between bone age and chronological age has been reported to have little effect on the initial acceleration of growth during GH therapy^6^. However, in children with ISS who are treated with GH, greater bone age delay has been associated with greater final height^7^.

In New Zealand, GH treatment is publically funded for growth hormone sufficient children with short stature^8^. In this study we examined the hypothesis that constitutional delay of growth is positively related with response to GH treatment and evaluated the relationship between age at the start of treatment, ethnicity, and sex on growth rate and increase in height in an unselected national cohort of New Zealand children and adolescents with short stature. As a secondary aim, we also evaluated the effect of bone age delay and GH treatment on change in predicted adult height.

## Methods

Review by the Multiregion Ethics Committee (Wellington, New Zealand) determined that ethics approval of this comprehensive audit was not required.

Anonymised patient data was provided by PHARMAC, the New Zealand agency that administers the GH program, from a database extending back to 1975^8^. All children receiving publicly funded GH therapy in New Zealand have applications processed centrally. GH was available for children with a diagnosis of ISS defined as a current or predicted adult height of more than 2 standard deviations (SDS) below the mean from 1975 to 1995, or more than 3 standard deviations (SDS) below the mean from 1995 to date, combined with a growth velocity for age that is less than the 25th percentile^9^, normal GH responses to at least 2 provocative tests, defined as a peak GH level > = 7 μg/L until 2010, and then > = 5 ug/L from 2010 to date, with no evidence of skeletal dysplasia or chronic illness. Turner Syndrome, chronic renal insufficiency and Prader Willi syndrome deficiency are separate funding categories, and were not included in this study. On-going funding is only approved by PHARMAC after assessing growth data submitted by local clinicians every 6 months, and requires a growth velocity greater than the 50^th^ percentile for age and pubertal status over a 12 month period^9^. GH treatment was started at 21 GH iu/m^2^/week and adjusted every 6 months to maintain this dose. Other growth promoting therapy such as oxandrolone or aromatase inhibitors were not available in New Zealand.

The patients' age, sex, ethnicity, pubertal status and bone age at baseline, serial 6 monthly height and weight measurements and annual bone age values were reported by the local hospitals caring for the patient, including (where available) the recorded adult height and adjusted mid parental height (aMPH). Height standard deviation score (HtSDS)^10^, weight SDS (WtSDS), BMI-SDS, and annual growth velocity SDS (GV-SDS)^9^ were calculated for a year before and the first 3 years after treatment. Adult HtSDS, predicted adult height (PAHSDS) at baseline and PAH at latest available time after the start of treatment were calculated using the British 1990 population as the reference measurement^10^. Bone ages were reported by the radiologists at each centre caring for the patients, based on the Greulich-Pyle standard^11^. Predicted adult height at the start of treatment, and at the oldest available age, was calculated for children 6 years of age or older, who had follow-up bone ages reported 12 months or more after the start of treatment. Final height was defined as the height achieved when GV was <2 cm/y and bone age was >14 y for girls and >16 y for boys. Insufficient birth weight and midparental height data were available for analysis (25/70). IGF-1 levels were not available for the majority of patients.

Data were analysed with SPSS v19 (SPSS Inc., Chicago, IL). The relationship between bone age and other variables was assessed by backwards multivariate linear regression. Changes over time were tested by ANOVA. P < 0.05 was considered to be significant.

## Results

79 children with ISS were identified from 1975 to 2013. Of these, 70 had a minimum of 1 year of growth data after starting GH; the remaining 9 children were excluded (Table 1). In these 70 children GH was given for a median of 3.2 years (range 1.0 to 9.7 years). The great majority showed a delayed bone age and were prepubertal. Boys were overrepresented (61.5%). The proportion of NZ Europeans (68.6%) was similar to the overall proportion in NZ (67.6%). 10% of the patients in this study were Maori, which is a little less than the estimate of 14.6% of the overall NZ population^12^.

**Figure 1:**
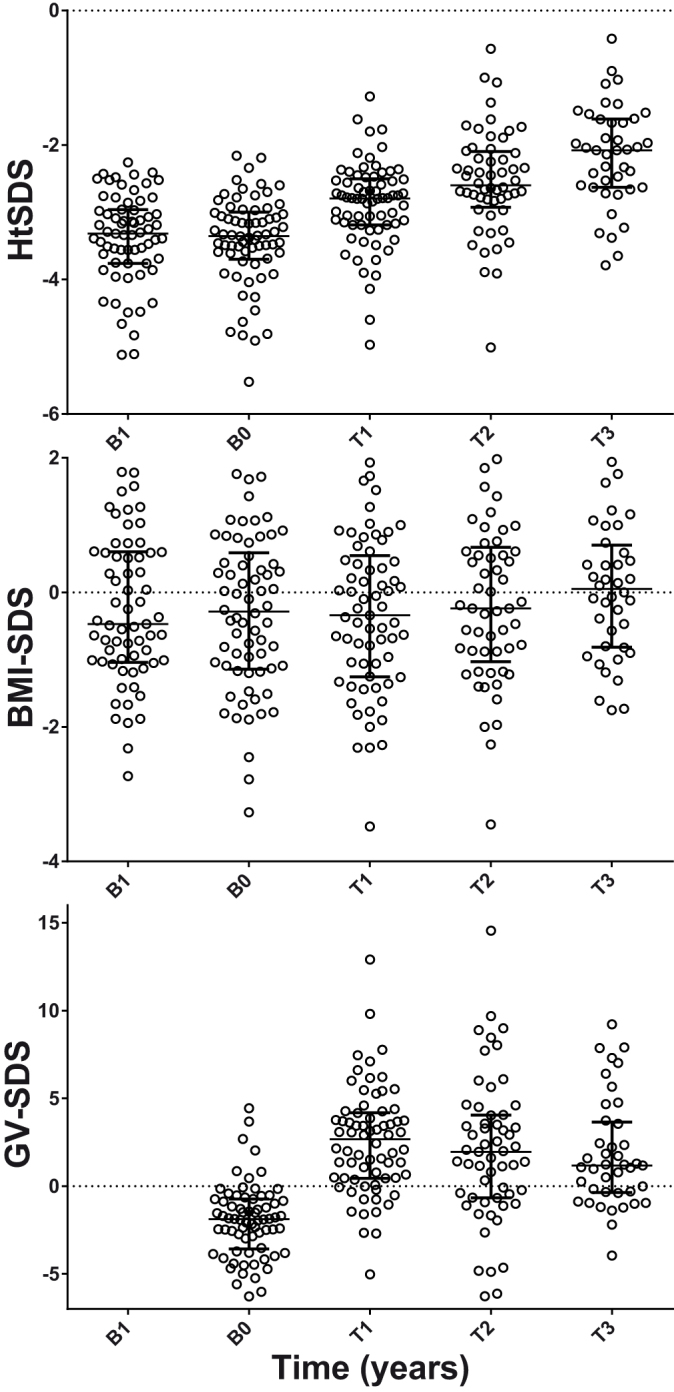
Time sequence of changes in Height SDS (Ht_SDS_), BMI_SDS_, and growth velocity SDS (GV_SDS_) before the start of GH treatment (B1 = 1 year before, and B0 = baseline) and for the first 3 years of treatment (T1 = year 1, T2 = year 2, T3 = year 3). Note the significant increase in Ht_SDS_ and GV_SDS_ in the first year of treatment, and continuing although slower increases thereafter. There was no significant change in BMI_SDS_. Bar and whiskers represent the medians and interquartile range.

**Figure 2:**
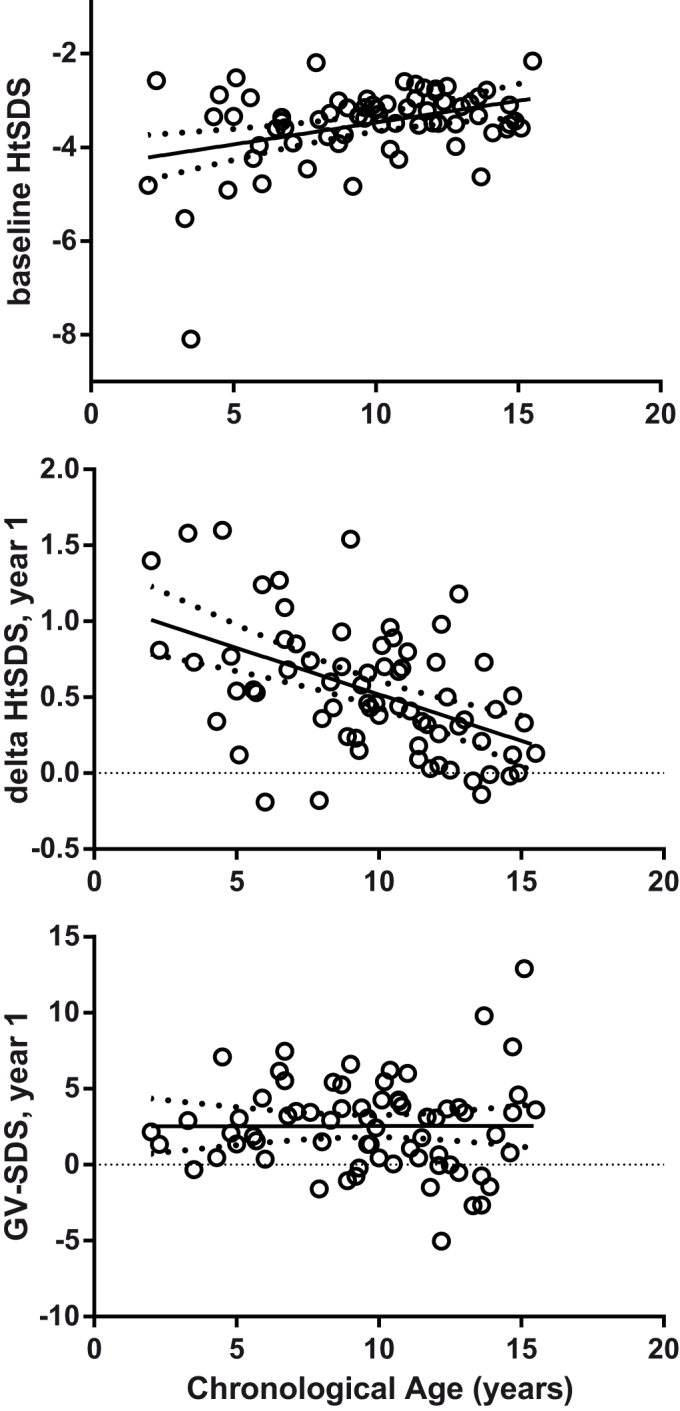
The relationship between chronological age at baseline, i.e. the start of GH treatment, baseline Ht_SDS_ (top panel), change in Ht_SDS_ in the first year (middle panel), and GV_SDS_ in first year of treatment (bottom panel). Note that younger children were shorter at the time of starting treatment but showed a much greater increase in HtSDS. Lines show the linear regression mean and 95% confidence intervals.

GH treatment was associated with an increase in both HtSDS and GV-SDS in the first year of treatment (p < 0.001, Figure 1), with no change in BMI-SDS. In the second and third year, there were smaller further increases in HtSDS, while GV-SDS remained significantly greater than baseline values. Younger age was associated with lower baseline HtSDS (r^2^ = 0.14, P = 0.002, Figure 2).

**Figure 3:**
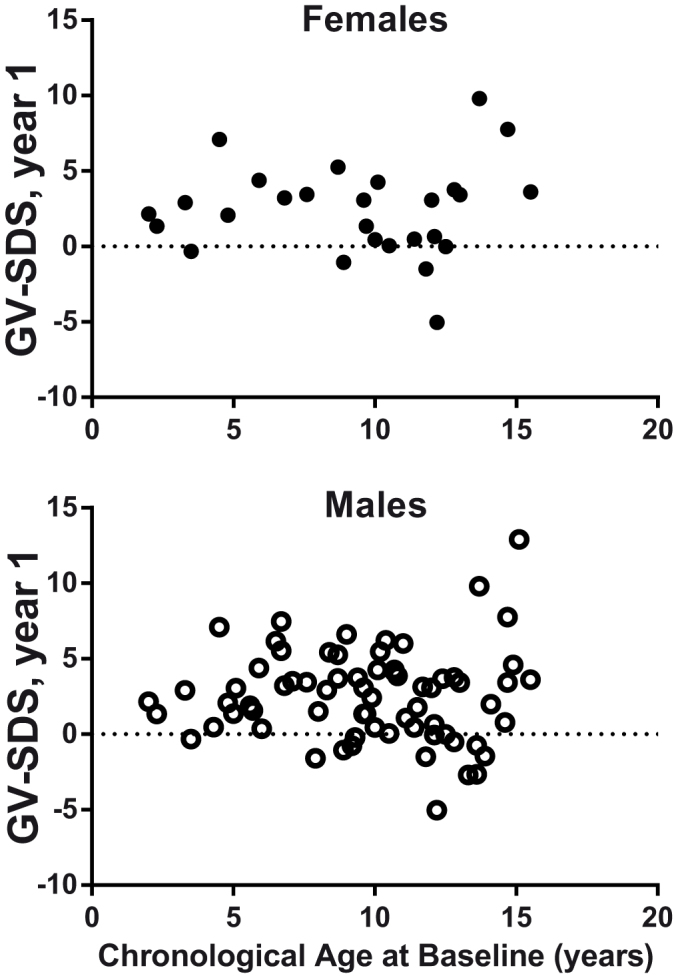
The relationship between chronological age at baseline and GV_SDS_ in first year of treatment for females (top panel) and males (bottom panel), showing a similar response to GH in boys and girls relative to age.

The increase in HtSDS in the first year was positively associated on multivariate analysis with younger age (r^2^ = 0.27, P < 0.001, Figure 2) and greater bone age delay (r^2^ = 0.05, P = 0.04), with no significant effect of sex, BMI-SDS or baseline HtSDS. In contrast, GV-SDS in the first year was not associated with any variable, including age, sex, bone age delay, baseline HtSDS, baseline GV-SDS or BMI-SDS (Figure 3). These results were not affected by excluding pubertal children from the analysis.

**Figure 4:**
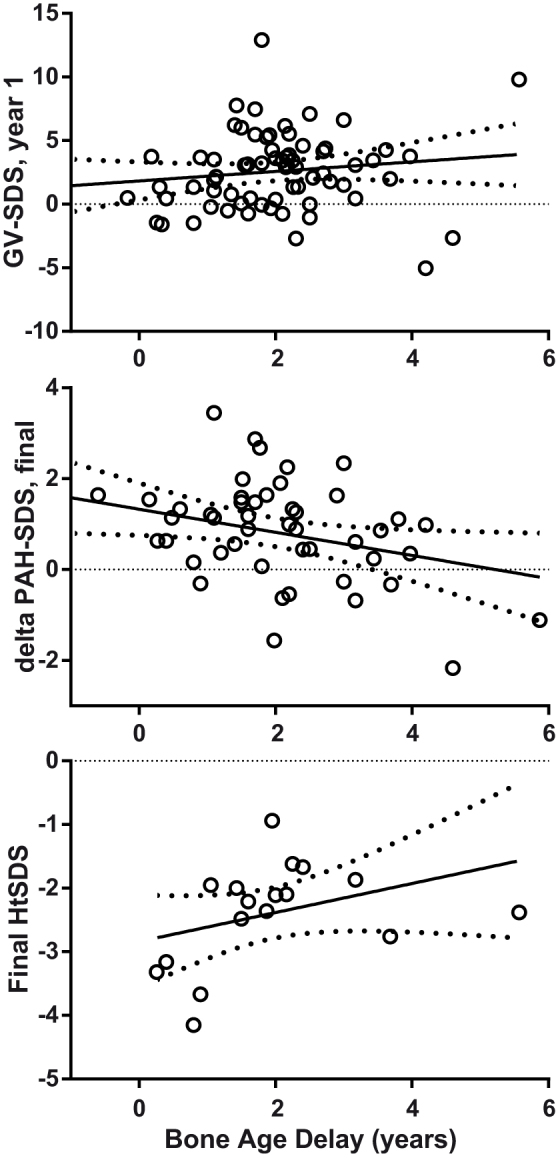
The relationship between bone age delay at baseline (years), and GV-SDS in the first year of treatment (top panel), change in PAH at the latest available time (middle panel), and final height SDS in a subset of 17 children (bottom panel). Note the significant apparent inverse correlation with delta PAH-SDS but positive trend for final height (r^2^ = 0.14, P = 0.15). There was no effect on GV-SDS in the first year of treatment. Lines show the linear regression mean and 95% confidence intervals.

Change in PAH-SDS during treatment was available for 48 children 6 years old or older at the start of treatment. PAH-SDS increased significantly compared with baseline (baseline −3.0 (−3.5, −2.3) vs final available −1.9 (−2.9, −1.4), P < 0.0001) at 14.4 years (12.0, 16.1). Reported bone ages advanced by 4.2 years (2, 6.4) over this interval, significantly more than the increase in chronological age of 3.2 years (1.8, 4.7) (P = 0.002, Mann Whitney U test). Consistent with this finding, the ratio of change in bone age to chronological age increased with greater initial bone age delay (r^2^ = 0.2, P = 0.001). Multivariate regression analysis suggested that the increase in PAH-SDS was inversely associated with bone age delay (r^2^ = 0.12, P = 0.03, Figure 4) and positively associated with delta HtSDS (r^2^ = 0.1, P = 0.02), with no significant effect of the age, sex, GV-SDS in the first year, BMI-SDS, baseline HtSDS, pubertal status or the timing of recruitment (<1995 vs > = 1995). In the small subset of cases for whom final height was available, there was no significant effect of bone age delay on final HtSDS (n = 17, r^2^ = 0.14, P = 0.15, Figure 4).

## Discussion

This study has demonstrated that while delayed bone age in an unselected group of children with short stature and growth hormone sufficiency was associated with a modestly greater initial growth response to GH treatment, greater delay was associated with a smaller increase in predicted final height after a median of 3 years of treatment. This effect likely reflects greater advancement in bone age during growth hormone treatment in those with the most severe bone age delay.

This study includes all children in NZ who received GH for ISS over a 38-year period. The children with ISS were shorter (median −3.4 SDS), than the median of −3 SDS reported in studies of GH treatment for ISS in Australia and Europe^4^. The cohort also differs from recent studies of ISS in that it includes children who were born small for gestational age. The children showed no change in height SDS in the two years before starting GH, with a marked and highly significant increase in height SDS and rate of growth in the first year of treatment. Some children failed to respond to this dose, as defined by a growth velocity less than the 50^th^ percentile for age, however, this would not have affected the primary analysis as GH supply was not stopped for lack of response in the first year of treatment. Importantly, there is evidence that a greater response to GH in the first year of treatment predicts a better long-term response overall to GH^4^,^6^. Although growth rates were less in the second and third year of treatment, overall growth rate remained both above average and better than pre-treatment rates, consistent with previous reports^4^.

Not surprisingly, the dose of GH can significantly affect both the initial increase in height and the long-term response in children with ISS and children with short stature who were born small for gestational age^13^,^14^. For example, Wit and colleagues found that a ‘low-dose' of 0.24 mg/kg/week (broadly equivalent to 6.7 mg/m^2^/week) was associated with smaller long-term increase in height (a mean of 5.4 cm vs 7.2 cm) than a ‘high-dose' of 0.37 mg/kg/week^13^. Interesting, a recent Australian study found a similar initial increase in HtSDS in patients with ISS treated with even lower doses that were titrated for effect (~4.5 mg/m2/day)^4^. The New Zealand target dose of 21 iu/m^2^/week corresponds broadly with 7 mg/m^2^/week, similar to the ‘low-dose' group in the study from Wit and colleagues, although it is important to appreciate that surface area based dosing will lead to relatively higher doses in smaller children than weight based dosing. Thus, the dose of GH used in New Zealand is in the lower half of the broad range used in previous studies.

The only factors that were significantly associated with greater improvement in height in the first year in the present study were younger age and greater bone age delay at the start of treatment. In contrast with previous studies^4^,^6^, we found no apparent effect of sex or baseline height or BMI-SDS on increase in height. Although the markedly better response in young children is similar to previous studies^4^,^15^, Ranke and colleagues reported that bone age delay was not associated with greater growth during GH therapy in the first year of treatment^4^, whereas it was associated with a small but significantly greater increase in HtSDS in the present study. This may reflect variation within a relatively small cohort or simply that the New Zealand patients had more severe ISS than international cohorts.

In contrast, there was no effect of age (or any other variable) at the start of treatment on subsequent growth velocity. Potentially, this difference could have been related to difficulty in accurately calculating growth velocity, particularly during puberty; however, no relationship was seen even when the analysis was restricted to children who were prepubertal at the time of starting treatment. A more likely explanation is that although increase in HtSDS is often taken as an index of rate of growth, the two are not the same, since the width of height SDS widens markedly with age, while conversely the rate of growth falls with age before puberty. This means that a given increase in growth rate has a greater effect on HtSDS at younger ages, supporting the importance of starting GH treatment as soon as possible in very short children. There was no effect of GH on BMI-SDS over time. It is possible that GH may have affected body composition as GH promotes the development of muscle over fat^16^, but specific tests such as dual-energy X-ray absorptiometry would be needed to show such a change.

In most published cohorts, approximately half of children have familial ISS^6^. There is some evidence that children with familial ISS may show a somewhat reduced response compared to non-familial ISS^6^. However, others have found very similar responses^4^. Unfortunately, in the present study midparental height data were not centrally collected by PHARMAC and so was not available for the majority of patients. Further, current definitions of ISS exclude children who were born small for gestational age, although it is a separate indication for GH treatment in many countries^2^. There is conflicting evidence for whether children who are small for gestational age respond similarly to children who were appropriate for gestational age at birth^17^,^18^. The New Zealand GH treatment programme was started well before this consensus, and so birth weight were not part of the entry criteria for ISS in New Zealand and so are not available for most children.

Predicted adult height increased significantly during GH treatment and was associated with greater increase in HtSDS in the first year but, intriguingly, greater delay of bone age appeared to be associated with less improvement. Consistent with this combination of greater initial increase in height SDS but reduced predicted adult height, we observed more rapid advancement of bone age in children with severe delay during GH therapy. Alternatively, this finding may imply that in routine use, the Bayley-Pinneau method used to predict future height over-adjusted for very large delays in bone age^19^. Consistent with this, the great majority of children in this study were prepubertal, and the accuracy of prediction of final height may be reduced before puberty, because of variation in the age of onset and tempo of puberty^20^. Against this, previous studies generally suggest that the Bayley-Pinneau method is reasonably accurate for short children; however, those studies have typically not involved children who are as short, young, and markedly delayed as in this study^21^,^22^. A limitation of the present study is that bone ages were collected on all children but were not centrally assessed, thus increasing inter-observer variation. Nevertheless, this study was a national cohort with all children being reviewed by 3 experienced paediatric endocrinologists before being approved for therapy. All had to satisfy the national criteria to receive a compassionate trial of GH therapy, and all data were collected prospectively. Thus there is a low likelihood of selection bias.

It is important to note that, by definition, reduced PAH is attenuation of a prediction, and should not be taken to denote a lack of benefit for final height. Rather, it merely suggests that the improvement in height would not be as much as initially ‘expected'. Overall, previous evidence suggests that delayed bone age is associated with improved final height in children with ISS^7^. Consistent with this, in the present study in the subset of children who had achieved final height, there was a trend to greater increase in final height with greater initial bone age delay. Further long-term follow-up is essential to resolve this issue. Regardless, this finding highlights the risk that our current routine clinical height prediction may be overly optimistic for children with severely delayed growth.

In conclusion, this study provides further evidence that GH is effective in increasing short-term growth in growth hormone sufficient children with short stature. The relative improvement in height was markedly better in younger children, supporting the importance of starting treatment early. Greater bone age delay was associated with greater initial improvement in height but apparently less improvement in predicted adult heights, highlighting risk that routine height prediction may be overly optimistic for children with severely delayed growth.

## Figures and Tables

**Table 1:** Demographic data and baseline auxological measurements

	n = 70
Sex (M:F)	43:27
Ethnicity (NZ European:Maori:Other)	48:7:15
Age, years	10.1 (7.0, 12.3)
Pubertal status (prepubertal:pubertal)	60:10
Bone age status (delayed:normal:advanced)	62:6:2
Bone age delay, years	1.9 (1.1, 3.0)
Height SDS	−3.4 (−3.7, −3.0)
Weight SDS	−2.6 (−3.2, −1.9)
BMI SDS	−0.29 (−1.1, 0.5)
GV SDS, before treatment	−1.9 (−3.6, −0.7)
Length of treatment, years	3.2 (1.8, 4.7)

Data are N, or median (25^th^, 75^th^ percentile). One patient did not have a baseline bone age.
